# Complement proteins and complement regulatory proteins are associated with age-related macular degeneration stage and treatment response

**DOI:** 10.1186/s12974-024-03273-7

**Published:** 2024-11-01

**Authors:** Alexander Kai Thomsen, Maria Abildgaard Steffensen, Jenni Martinez Villarruel Hinnerskov, Amalie Thomsen Nielsen, Henrik Vorum, Bent Honoré, Mogens Holst Nissen, Torben Lykke Sørensen

**Affiliations:** 1 Department of Ophthalmology, Zealand University Hospital, Sygehusvej 10, Roskilde, 4000 Denmark; 2 Department of Clinical Medicine, University of Copenhagen, Copenhagen, Denmark; 3 Department of Immunology and Microbiology, University of Copenhagen, Copenhagen, Denmark; 4 Department of Clinical Medicine, Aalborg University Hospital, Aalborg, Denmark; 5 Department of Ophthalmology, Aalborg University Hospital, Aalborg, Denmark; 6 Department of Biomedicine, Aarhus University, Aarhus, Denmark

**Keywords:** Neovascular age-related macular degeneration, Intermediate age-related macular degeneration, Complement, Complement regulatory proteins, T cells, Monocytes, Treatment response, Inflammation, CFH, ARMS2, Genetics

## Abstract

**Background:**

Dysregulation of the complement system is involved in development of age-related macular degeneration (AMD). The complement cascade is regulated by membrane bound complement regulatory proteins (Cregs) on mononuclear leukocytes among others. This study aims to investigate systemic complement proteins and Cregs in AMD stages and their association with treatment response in neovascular AMD (nAMD).

**Methods:**

In this clinical prospective study, treatment-naïve patients with nAMD, intermediate AMD (iAMD) and healthy controls were recruited and systemic complement proteins C3, C3a and C5a were investigated with electrochemiluminescence immunoassays, and Creg expression (CD35, CD46 and CD59) on T cells (CD4 + and CD8+) and monocytes (classical, intermediate and non-classical) investigated with flow cytometry. Treatment response in nAMD patients was evaluated after loading dose and after one year, and categorized as good, partial or poor. Complement proteins and Creg expression levels were compared between healthy controls, iAMD and nAMD, as well as between good, partial and poor nAMD treatment response groups. Polymorphisms in the CFH and ARMS2 genes were analyzed and compared to complement proteins and Creg expression levels in nAMD patients.

**Results:**

One hundred patients with nAMD, 34 patients with iAMD and 61 healthy controls were included. 94 nAMD patients completed the 1-year follow-up. Distribution of treatment response in nAMD was 61 (65%) good, 26 (28%) partial, and 7 (7%) poor responders. The distribution of 1-year treatment response was 50 (53%) good, 33 (36%) partial, and 11 (11%) poor responders. The concentrations of systemic C3, C3a, and the C3a/C3-ratio were significantly increased in patients with nAMD compared to healthy controls (*P* < 0.001, *P* = 0.002, and *P* = 0.035, respectively). Systemic C3 was also increased in iAMD compared to healthy controls (*P* = 0.031). The proportion of CD46 + CD4 + T cells and CD59 + intermediate monocytes were significantly decreased in patients with nAMD compared to healthy controls (*P* = 0.018 and *P* = 0.042, respectively). The post-loading dose partial treatment response group had significantly lower concentrations of C3a and C5a compared to the good response group (*P* = 0.005 and *P* = 0.042, respectively). The proportion of CD35 + monocytes was significantly lower in the 1-year partial response group compared to the 1-year good response group (*P* = 0.039). High-risk CFH genotypes in nAMD patients was associated with increased C3a, C3a/C3-ratio, and expression levels of CD35 + CD8 + T cells and CD46 + classical monocytes, while expression level of CD46 + non-classical monocytes was decreased.

**Conclusion:**

Elevated concentrations of systemic complement proteins were found in patients with iAMD and nAMD. Decreased Creg expression levels were found in patients with nAMD. Partially responding nAMD patients had a dysregulated complement system and Cregs compared to good responders.

**Supplementary Information:**

The online version contains supplementary material available at 10.1186/s12974-024-03273-7.

## Introduction

Age-related macular degeneration (AMD) is a leading cause of visual impairment and blindness in the elderly [1]. The preliminary stage of the disease is intermediate AMD (iAMD) characterized by macular drusen, but few clinical symptoms. Two types of late-stage AMD can develop from iAMD, called neovascular AMD (nAMD) and geographic atrophy. Neovascular AMD is fast-progressing and can cause symptoms of metamorphopsia and central scotomas within weeks to months [2]. Treatment of nAMD consists of repeated injections with an anti-vascular endothelial growth factor (VEGF) antibody, that can stabilize the disease and, in some cases, even reverse the symptoms completely. However, treatment response differs greatly between individuals and some patients will continue to experience visual deterioration [3]. Thus, VEGF might not be the only mediating factor for neovascularization secondary to AMD. In patients with insufficient response to anti-VEGF treatment, it may be beneficial to add an additional therapy targeting a different pathway [4].

The cause of AMD is multifactorial and is not yet completely elucidated. Environmental factors, genetics and chronic low-grade inflammation play significant roles in the pathophysiology [5–8]. Age-related chronic inflammation and dysregulated immune responses with involvement of both the innate and adaptive immune systems have been shown to increase the risk of developing AMD [9]. Alterations in the complement system have previously been shown to be associated with development and stages of AMD [10–15]. The complement system is responsible for enhancing the ability of antibodies and phagocytic cells to clear pathogens and damaged cells. It functions through a cascade of protein activations that lead to pathogen opsonization, promotion of inflammation, and direct lysis of pathogens by forming the membrane attack complex (MAC). Complement protein C3 is central in this cascade by cleavage into C3a, which acts as an anaphylatoxin activating multiple inflammatory pathways, and C3b, which acts as an opsonin and forms part of the MAC. C3b is also involved in creating C5 convertase that cleaves C5 into C5a and C5b further downstream in the complement cascade. C5a, like C3a, is an anaphylatoxin, which activates phagocytosis in immune cells like monocytes [16].

Dysregulation of complement regulatory proteins (Cregs) has also been found in patients with nAMD [17–19]. CD35 (Complement Receptor 1) and CD46 (Membrane Cofactor Protein) are involved in regulation of complement activation on T cells and monocytes [20]. CD59 (MAC inhibiting protein) inhibits the formation of MAC, thus protecting cells from lysis [20]. CD11b (Integrin α-M) is expressed on monocytes, among other cells, and facilitates adhesion and migration in inflammatory sites. Furthermore, CD11b is part of Complement Receptor 3 involved in complement mediated phagocytosis [21, 22].

Genetic predisposition is a key factor in AMD risk and more than half of AMD heritability is associated with genes related to the complement cascade [16, 23]. Polymorphisms in the complement factor H (CFH) gene has been extensively studied and the single nucleotide polymorphism (SNP) CFH rs1061170 has been shown to be strongly associated with AMD [24–28]. The polymorphism Age-Related Maculopathy susceptibility 2 (ARMS2) rs10490924 is also a major genetic risk factor for AMD, although the function of the ARMS2 protein remains largely unknown [24–27, 29].

This study aims to investigate the association between systemic complement protein concentrations and AMD stage (healthy controls, iAMD and nAMD), as well as the association between systemic Creg expression levels on mononuclear leukocytes (T cells and monocytes) and AMD stage. Because of previous findings of dysregulated complement proteins and Cregs in nAMD, we further sought to investigate the association of these proteins and treatment response in nAMD patients. Furthermore, we explored whether these complement proteins and Cregs were associated with the risk polymorphisms CFH rs1061170 and ARMS2 rs10490924 in nAMD patients. This may further our understanding of AMD pathophysiology and potentially reveal new therapeutic targets.

## Methods

### Study Design and participants

The Danish Neovascular Age-Related Macular Degeneration and Treatment Response (DANEART) study is a prospective cohort study investigating immunological profiles of patients with nAMD, iAMD and healthy controls. The study is a single-center study conducted at the Department of Ophthalmology, Zealand University Hospital, Denmark approved by the Regional Committee of Ethics in Research of the Region of Zealand, Denmark (journal no: SJ-768) and performed in adherence with the Declaration of Helsinki. Verbal and written informed consent were obtained from all participants prior to inclusion.

Treatment-naïve patients with nAMD, patients with iAMD and healthy controls were consecutively enrolled in this study. Exclusion criteria were age younger than 60 years, inflammatory, autoimmune, cancer and infectious diseases, use of immunomodulating treatment, active smoking, plasma C-reactive protein > 15 mg/L, vision-affecting disorders other than nAMD and iAMD, and previous treatment for nAMD.

Healthy controls and patients with iAMD were examined at baseline, while patients with nAMD were examined at baseline and two follow-up examinations. Patients with nAMD had diagnosis and disease severity evaluated at baseline, while progression was evaluated at follow-ups post-loading dose and after one year. All nAMD patients were treated according to the observe-and-plan regimen with aflibercept as per Danish national guidelines [30].

### Clinical investigations and medical interview

All participants were examined by a retinal specialist for best corrected visual acuity (BCVA), slit-lamp biomicroscopy, color fundus photography, spectral domain optical coherence tomography (OCT), and OCT angiography. Diagnosis of nAMD and iAMD was based on multimodal imaging. Participants were interviewed regarding their medical history, medications and smoking habits, which was crosschecked with the electronic health records.

### Grading disease and treatment response

Healthy controls were examined with the same thorough examination as patients to confirm they were indeed ophthalmologically normal. AMD stage was classified according to the Beckman criteria [31]. Eyes with presence of large drusen (diameter > 125 μm) or pigmentary abnormalities associated with at least medium drusen (> 65–125 μm) were classified as iAMD. Eyes with neovascularization and exudative changes on multimodal imaging were classified as nAMD.

OCT scans were graded to determine disease severity and treatment response in patients with nAMD. These scans were evaluated for presence of intra- and subretinal fluid, and central retinal thickness (CRT). Patients with nAMD were classified according to treatment response post-loading dose and after one year, according to criteria previously described [3, 4]. In brief, good response was classified as total regression of retinal fluid, partial response as persistence of retinal fluid and a reduction of CRT, and poor response as persistence of retinal fluid and unchanged or increased CRT (Table 1). The eye with nAMD was chosen as the study eye. In cases of bilateral nAMD, the right eye was chosen.

**Table 1:** Definitions of treatment responses in nAMD patients evaluated on optical coherence tomography scans

	Treatment response
Good	Total regression of IRF and SRF
Partial	Persistence of IRF and/or SRF and reduction of CRT
Poor	Persistence of IRF and/or SRF and unchanged or increased CRT

IRF: Intraretinal fluid

SRF: Subretinal fluid

CRT: Central retinal thickness

### Blood sampling

Blood sampling and flow cytometry were performed at baseline. Blood was sampled from the antecubital vein in tubes coated with ethylenediamine-tetraacetic acid (EDTA) for flow cytometry and complement protein assays, as well as lithium-heparin with gel for plasma C-reactive protein.

### Flow cytometry

**Fig. 1:**
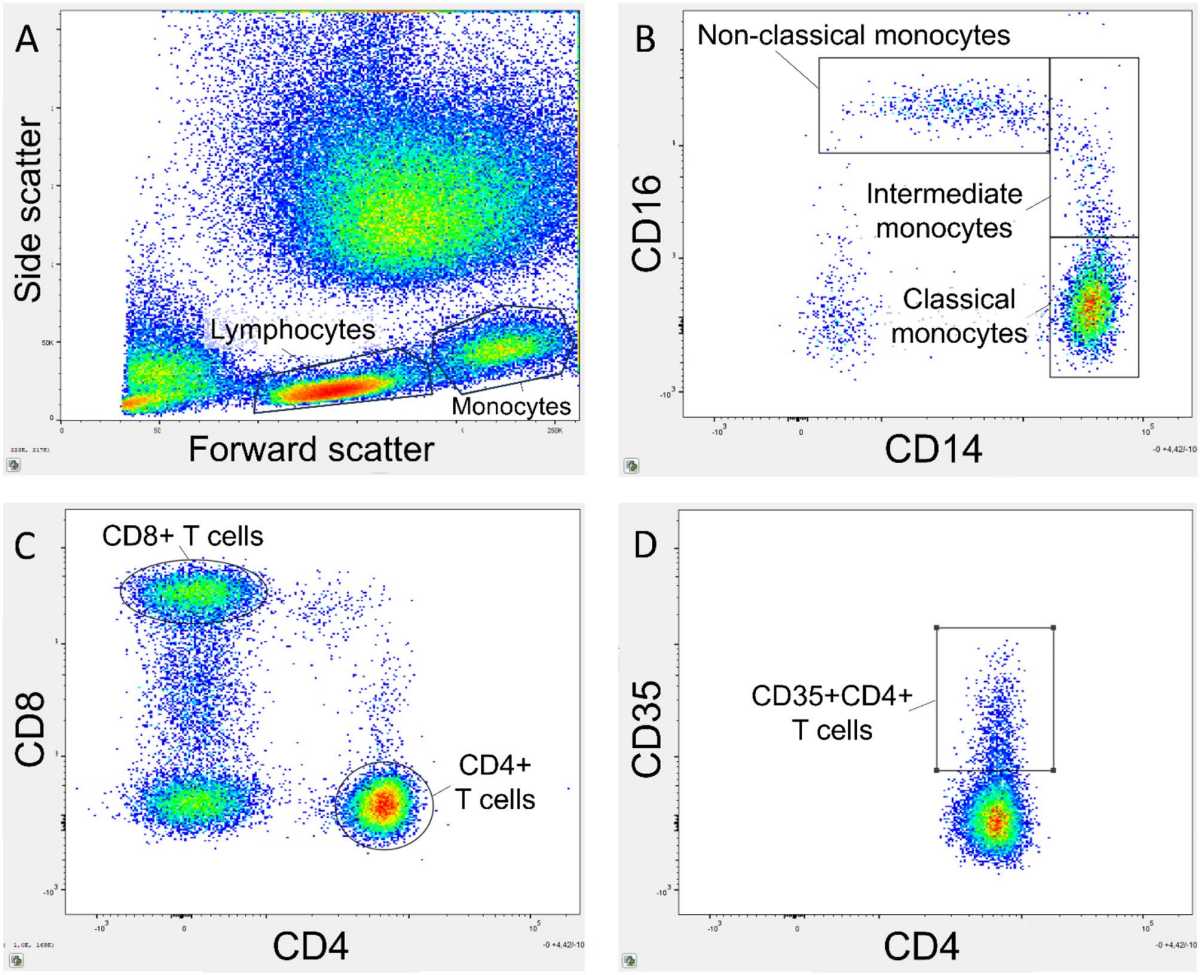
Flow cytometry gating strategy. (**A**) Lymphocytes and monocytes were identified on the forward-side-scatter. (**B**) Monocyte subsets were identified as classical (CD14^high^CD16^low^), intermediate (CD14^high^CD16^high^), and non-classical (CD14^low^CD16^high^). (**C**) CD4 + T cells and CD8 + T cells were identified among the lymphocytes. (**D**) Complement regulatory proteins were gated on the leukocyte subgroups with Boolean sequences, in this example CD35 on CD4 + T cells

Flow cytometry preparations were initiated within 4 h of phlebotomy. Leukocyte count was performed on Sysmex KX-21NTM (Sysmex Corporation, Kobe, Japan) to calculate blood volume sufficient to obtain 1.0 × 10^6^ leukocytes for analysis. To lyse erythrocytes a 1% lysis buffer (BioLegend, San Diego, CA, USA) was added to the blood sample and stored at room temperature in the dark for 10 min. Cells were washed three times in a process of adding BD FACS Flow isotonic buffer, centrifugation at 500 × g for five minutes, followed by decantation of the supernatant. The isolated leukocytes were then resuspended in isotonic buffer and monoclonal fluorescent antibodies were added (Supplementary Table 1, Additional File 1) and incubated for 20 min at room temperature in the dark. The stained leukocytes were washed and resuspended in isotonic buffer a last time before being analyzed on the BD FACS Canto II flow cytometer (BD Bioscience, San Jose, CA, USA) with a gating size of 100.000 singlet cells analyzed per sample. The flow cytometry data was analyzed with FlowJo software (Tree Star, Ashland, OR, USA, v.10.10.0). Gating strategy consisted of identifying lymphocytes and monocytes on a forward-side scatter plot, followed by singlet cells on a forward area-forward height scatter plot. Lymphocytes were gated for CD4 and CD8 to identify CD4 + T cells and CD8 + T cells. These cells were then gated for the surface membrane Cregs CD35, CD46, and CD59. Monocytes were gated for CD16 and CD14 to identify classical (CD14^high^CD16^low^), intermediate (CD14^high^CD16^high^) and non-classical (CD14^low^CD16^high^) monocytes. Monocytes and monocyte subgroups were gated for CD35, CD46, CD59 and CD11b (Fig. 1).

### Cytokine assays

The plasma concentrations of complement proteins C3, C3a, and C5a were quantified with immunoassays. The EDTA coated tubes for the assays were centrifuged at 1500 × g for 15 min at 20 °C immediately after phlebotomy. The plasma was isolated, frozen at -80 °C within 1 h and analyzed a different day. The assays were performed with the commercially available electrochemiluminescence R-plex immunoassays (Mesoscale Discovery, Rockville, MD, USA). Blood samples were thawed and analyzed in duplicate according to manufacturer guidelines. The specific assays used, can be found in Supplementary Table 2, Additional File 1. The C3a/C3-ratio was calculated as a measure of complement activation [32].

### Genotyping

Genotyping of the SNPs CFH rs1061170 and ARMS2 rs10490924 was performed on EDTA full blood from nAMD patients. These tubes were frozen at -80 °C immediately after phlebotomy and analyzed a different day. Genomic DNA extraction and SNP analyses were performed by BioXpedia, Denmark. Using the Fluidigm GT192.24 Dynamic Array Integrated Fluidic Circuit (Fluidigm Corp., San Fransisco, CA, USA) according to the manufacturer’s protocol. The data was analyzed with the Fluidigm SNP Genotyping analysis software v.4.5.1 with standard settings.

### Statistics

Statistical analysis was performed with R software version 4.2.3 (R Foundation for Statistical Computing, Vienna, Austria). Normally distributed data is reported as mean and standard deviation (SD). Analysis of covariance (ANCOVA) was performed to evaluate the differences of complement proteins and Cregs according to diagnosis and treatment response groups. Healthy controls were chosen as reference group in the diagnosis analysis, and good responders as reference group in the treatment response analyses. All ANCOVA analyses were adjusted for age, as age-related changes of immunosenescence is a well described phenomenon [9], and smoking (never or previous smoker) as it has been shown that smoking increases systemic proinflammation [33]. Logarithmic transformation was applied as appropriate in cases of a positive skewness to fit assumption of normality. Correlation networks were created showing nodes, representing the complement proteins and Cregs, connected by edges (lines) of statistically significant correlations. The thickness of the edges indicates the absolute correlation coefficient with a threshold of > 0.4. These networks can help visualize the complex correlations of the complement cascade and regulation. Association between genotypes and complement proteins, and Cregs were performed using Welch two sample t-test. A *P* value < 0.05 was interpreted as statistically significant. As the analyzed parameters are related, non-independent factors, a statistical adjustment for multiple testing might be too conservative and was not performed [34].

## Results

### Study Population

A total of 100 patients with nAMD, 34 patients with iAMD and 61 healthy controls were included. Healthy controls were significantly younger than nAMD patients, however age was adjusted for in subsequent analyses. There were no significant differences in other participant characteristics (Table 2).

**Table 2:** Patient characteristics

	Diagnosis			
	Healthy Controls (*n* = 61)	iAMD (*n* = 34)	nAMD (*n* = 100)	*P* value
Age, years, mean (SD)	73 (7)	75 (8)	80 (6)	< 0.001
Hypertension, n (%)	30 (49)	19 (56)	64 (64)	0.17
Hypercholesterolemia, n (%)	17 (28)	5 (15)	28 (28)	0.28
Cardiovascular disease, n (%)	20 (33)	8 (24)	37 (37)	0.37
Type 2 diabetes, n (%)	7 (12)	1 (3)	6 (6)	0.27
Smoking status, n (%)				
Never	27 (44)	17 (50)	38 (38)	0.43
Previous	34 (56)	17 (50)	62 (62)	

BCVA: Best corrected visual acuity

ETDRS: Early treatment of diabetic retinopathy study

Of the 100 nAMD patients, 94 completed the 1-year follow-up. Five participants died and one was excluded due to inability of following the treatment plan. The nAMD patients treated with anti-VEGF injections responded differently to treatment. The distribution of post-loading dose treatment response in patients with nAMD was 61 (65%) good, 26 (28%) partial, and 7 (7%) poor responders. The distribution of 1-year treatment response was 50 (53%) good, 33 (36%) partial, and 11 (11%) poor responders. Baseline median (IQR) visual acuity of initial treatment response was 64 (23), 62.5 (19.5), and 58 (13) ETDRS letters for good, partial and poor responders, respectively (*P* = 0.61). Mean (SD) number of injections were 5.8 (1.4), 6.8 (1.9) and 6.3 (1.8) for good, partial and poor responders after one year (*P* = 0.14).

### Complement proteins and Cregs Association to diagnosis

**Fig. 2:**
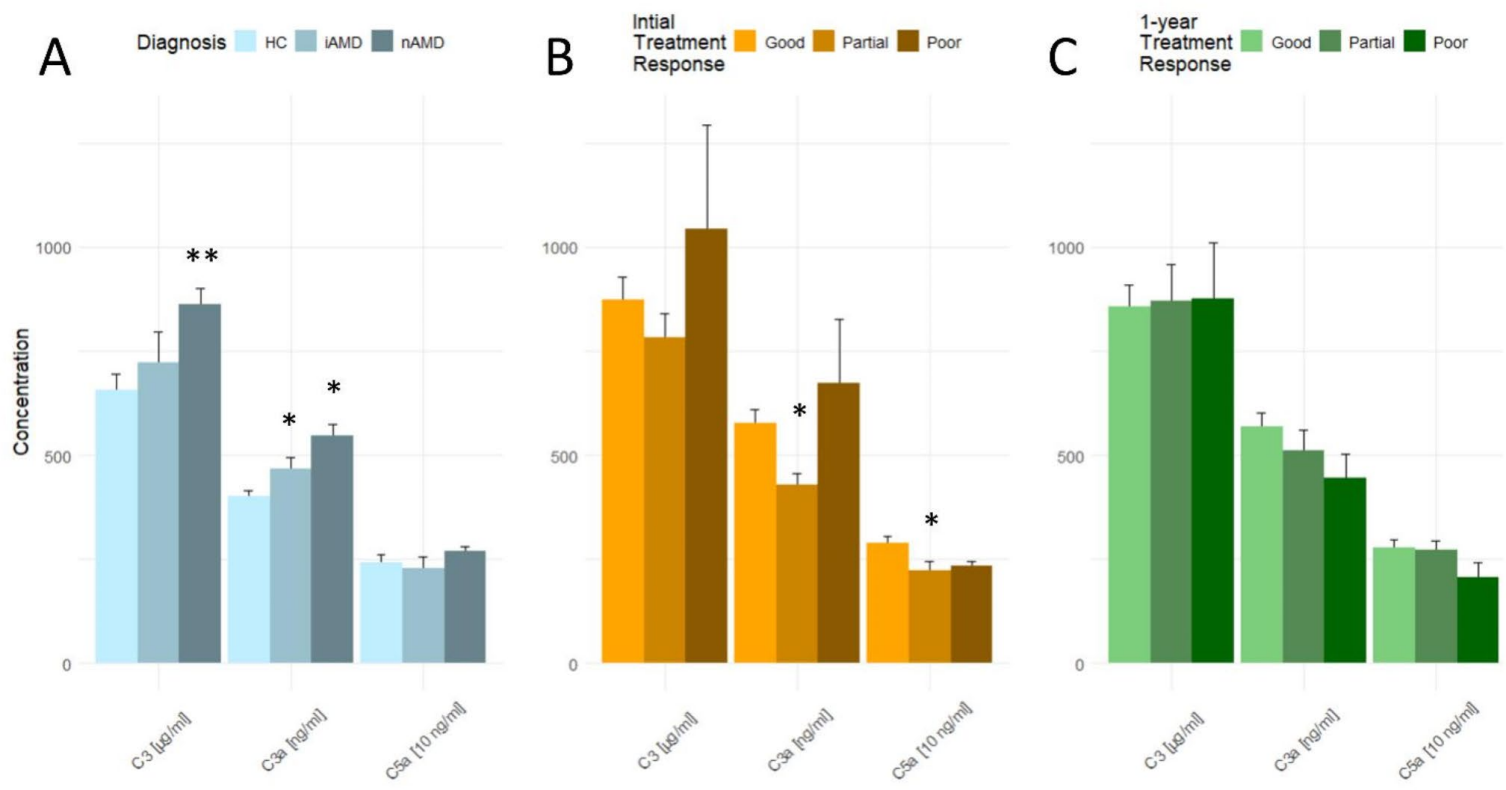
Concentration of complement proteins according to (**A**) Diagnosis, (**B**) Initial treatment response of nAMD patients, (**C**) 1-year treatment response of nAMD patients. HC = healthy controls; iAMD = intermediate AMD; nAMD = neovascular AMD. * *P* < 0.05; ** *P* < 0.001 compared to reference group (healthy controls or good treatment response)

**Fig. 3:**
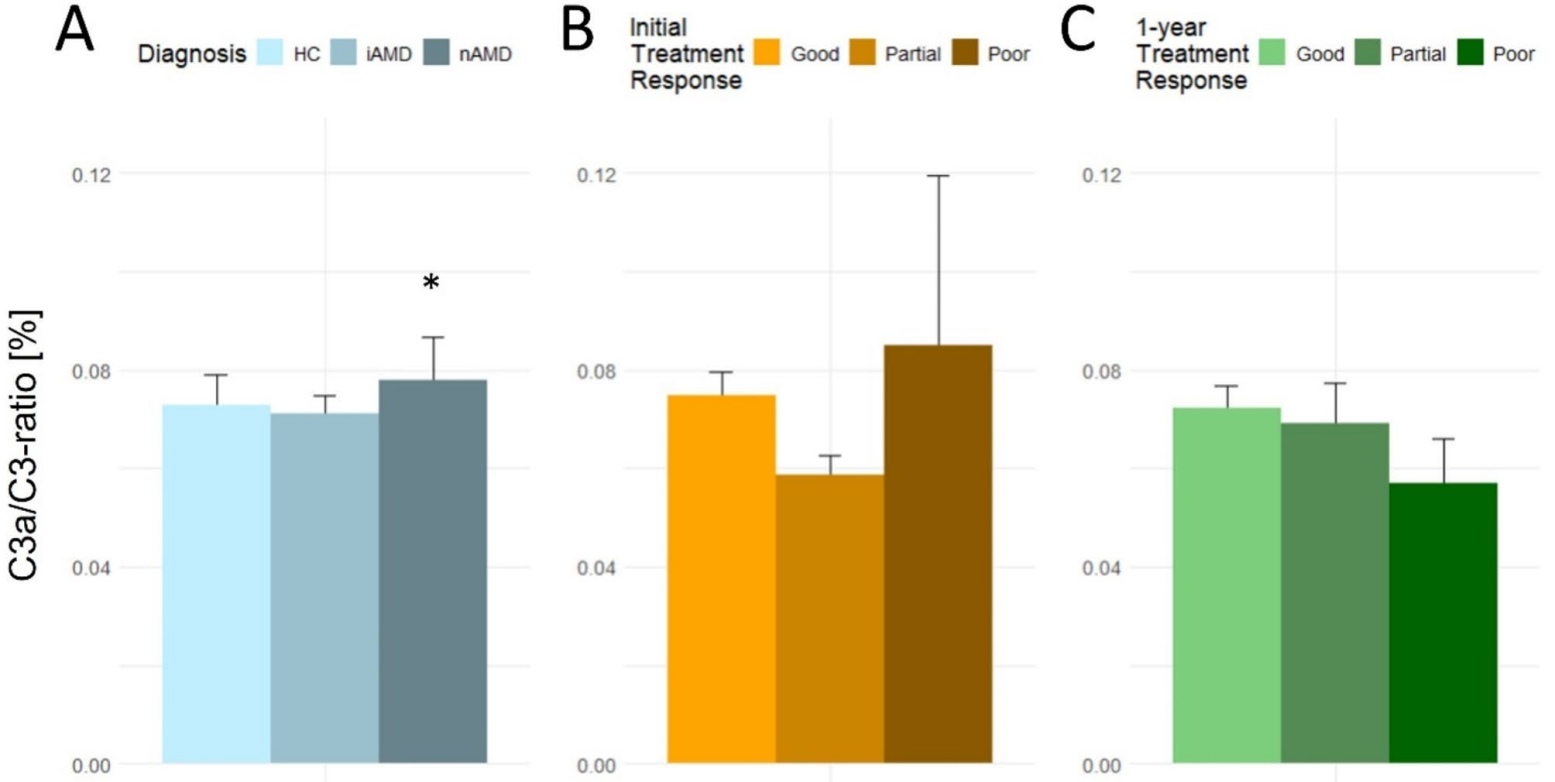
Complement C3a/C3-ratio [%] according to (**A**) Diagnosis, (**B**) Initial treatment response of nAMD patients, (**C**) 1-year treatment response of nAMD patients. HC = healthy controls; iAMD = intermediate AMD; nAMD = neovascular AMD. * *P* < 0.05; ** *P* < 0.001 compared to reference group (healthy controls or good treatment response)

The concentration of complement protein C3 differed significantly between healthy controls and patients with nAMD (Fig. 2A). The concentration of C3 in nAMD patients was 862 µg/ml (SD, 375), which was significantly higher than 656 µg/ml (SD, 262) in healthy controls (*P* < 0.001). Concentrations of C3a also differed significantly between nAMD and iAMD compared to healthy controls. Patients with nAMD had a C3a concentration of 548 ng/ml (SD, 238) significantly higher than 401 ng/ml (SD, 100) of healthy controls (*P* = 0.002). Likewise, iAMD patients had a significantly higher C3a concentration of 467 µg/ml (SD, 124) compared to healthy controls (*P* = 0.031). There was no significant difference between C3 in healthy controls and iAMD patients, or between C5a between the treatment groups. The mean (SD) C3a/C3-ratio in nAMD patients was 0.078% (0.009%), which was significantly higher than 0.073% (0.006%) in healthy controls (*P* = 0.035) (Fig. 3A).

**Fig. 4:**
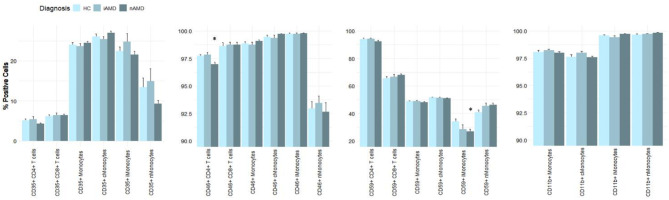
Proportion of complement regulatory proteins according to diagnosis. HC = healthy controls; iAMD = intermediate AMD; nAMD = neovascular AMD; cMonocytes = classical monocytes; iMonocytes = intermediate monocytes; nMonocytes = non-classical monocytes. * *P* < 0.05; ** *P* < 0.001 compared to reference group (healthy controls)

The expression of the Creg CD46 was slightly, but significantly, decreased on CD4 + T cells in nAMD patients compared to healthy controls. The proportion of CD46 + CD4 + T cells was 97.0% (SD, 1.9) in nAMD patients and 97.7% (SD, 1.0) in healthy controls (*P* = 0.018). The proportion of CD59 on intermediate monocytes in nAMD patients was 26.8% (SD, 17.5), which was significantly lower than 34.2% (SD, 15.1) in healthy controls (*P* = 0.042). Healthy controls did not differ significantly from iAMD or nAMD patients in Creg proportion of CD35, CD46, CD59 and CD11b on T cells or monocytes, other than the two aforementioned (Fig. 4).

**Fig. 5:**
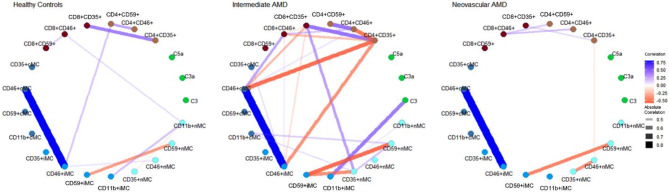
Correlation network of complement proteins and complement regulatory proteins according to diagnosis, showing statistically significant correlations (*P* < 0.05) with a threshold of absolute correlation coefficient > 0.4

The correlations of complement proteins and Cregs seems to differ between healthy controls, iAMD and nAMD, showing unique phenotypes of the complement and complement regulatory systems. Especially patients with iAMD seemed to have a more complex network of correlations between complement proteins and Cregs, while healthy controls and nAMD had fewer significant correlations (Fig. 5).

### Complement proteins and Cregs Association to initial treatment response of nAMD patients

Concentrations of complement proteins C3a and C5a differed in nAMD patients’ initial treatment response group. Patients with a partial initial treatment response had a C3a concentration of 429 ng/ml (SD, 127), which was significantly lower than the good initial treatment response with a concentration of 576 ng/ml (SD, 252) (*P* = 0.005). Patients in the partial initial treatment response group also had a significantly lower concentration of C5a compared to good initial treatment response (mean (SD) concentration of 22.2 ng/ml (11.2) and 29.0 ng/ml (11.6), respectively, *P* = 0.010). There was a trend toward higher concentrations of C3 and C3a in patients with poor initial treatment response, however not statistically significant. No significant difference was found between C5a concentrations of poor and good initial responders (Fig. 2B). No significant difference in C3a/C3-ratio was found between treatment response groups (Fig. 3B).

**Fig. 6:**
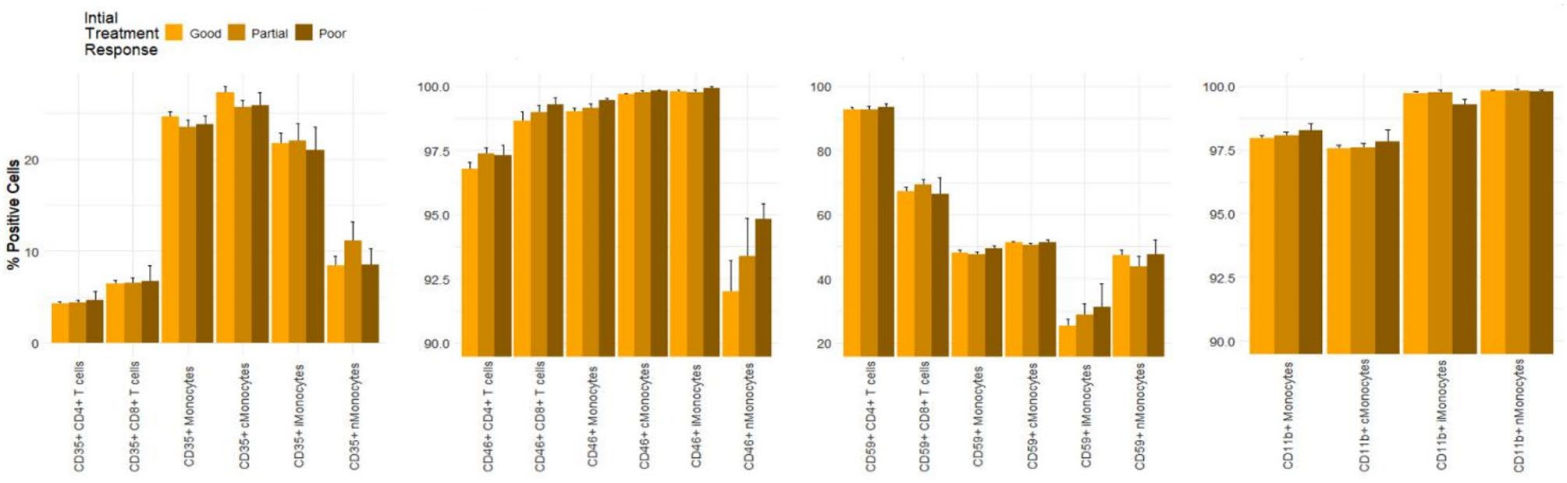
Proportion of complement regulatory proteins according to initial treatment response in neovascular AMD patients. cMonocytes = classical monocytes; iMonocytes = intermediate monocytes; nMonocytes = non-classical monocytes. * *P* < 0.05; ** *P* < 0.001 compared to reference group (good treatment response)

A tendency of a higher proportion of CD46 + non-classical monocytes in poor responders appears in Fig. 6, however not significant (*P* = 0.46). The expression levels of the Cregs CD35, CD46, CD59 and CD11b did not differ significantly between initial treatment response groups on CD4 + T cells, CD8 + T cells, monocytes or monocyte subgroups (Fig. 6).

**Fig. 7:**
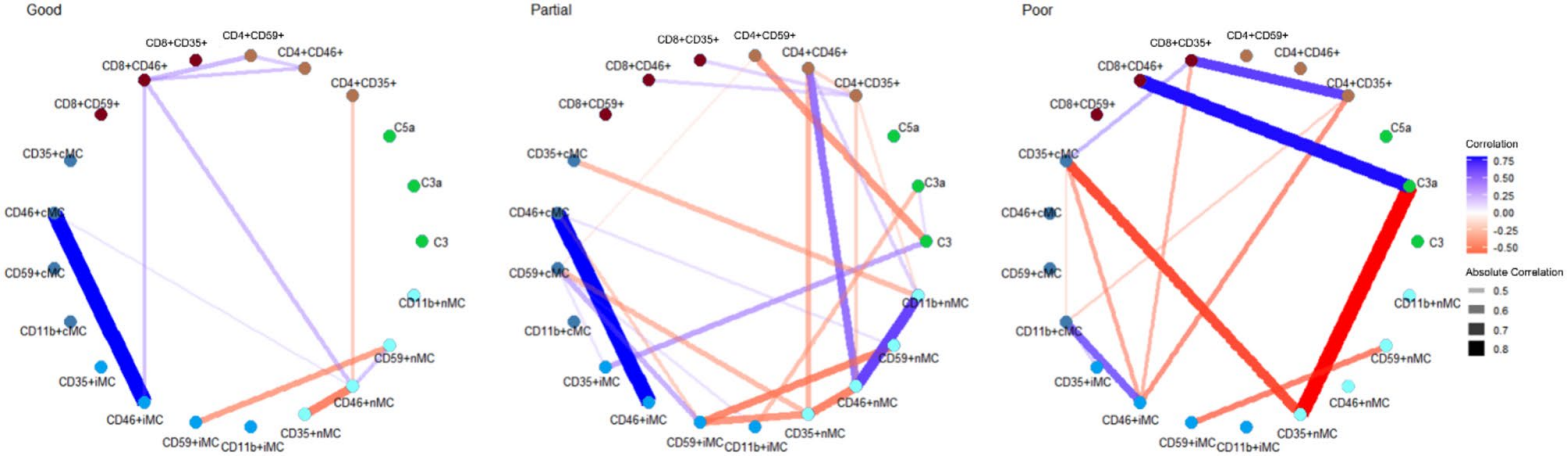
Correlation network of complement proteins and complement regulatory proteins according to initial treatment response in neovascular AMD patients, showing statistically significant correlations (*P* < 0.05) with a threshold of absolute correlation coefficient > 0.4

The correlation between complement proteins and Cregs seemed to show intergroup differences between each initial treatment response group. Noticeably, complement proteins seemed to play a more central role in partial and poor responders compared to good responders. Partial responders have the most complex network with the most significant correlations (Fig. 7).

### Complement proteins and Cregs Association to 1-year treatment response of nAMD patients

Complement proteins and C3a/C3-ratio did not differ significantly between 1-year treatment groups (Figs. 2C and 3C).

**Fig. 8:**
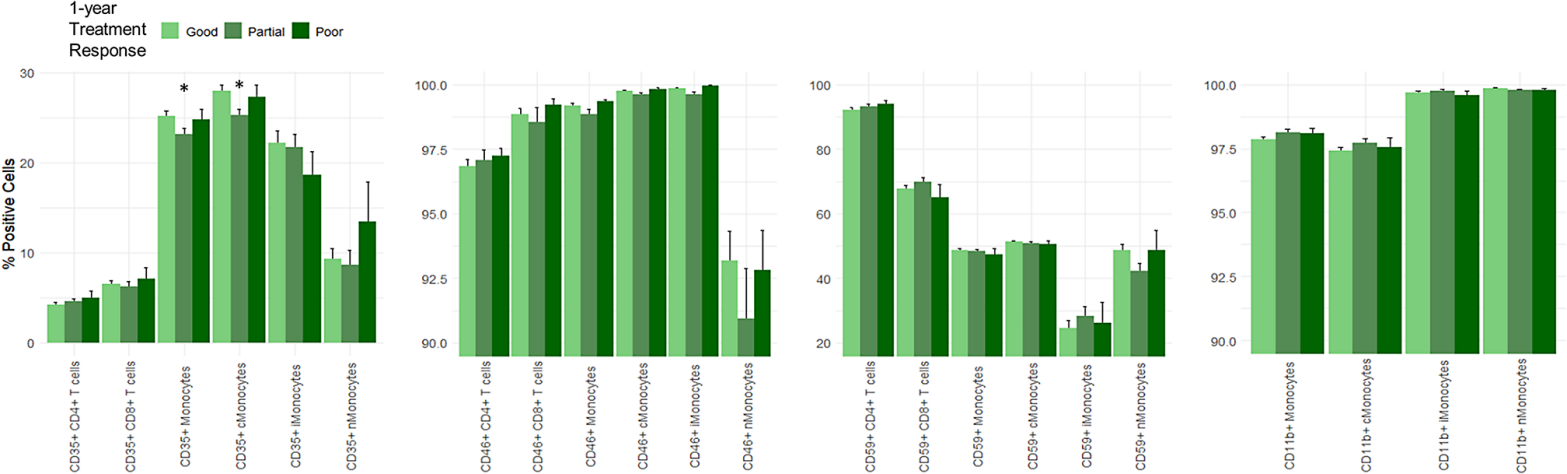
Proportion of complement regulatory proteins according to 1-year treatment response in neovascular AMD patients. cMonocytes = classical monocytes; iMonocytes = intermediate monocytes; nMonocytes = non-classical monocytes. * *P* < 0.05; ** *P* < 0.001 compared to reference group (good treatment response)

Patients with partial 1-year treatment response differed significantly in proportion of CD35 + monocytes and CD35 + classical monocytes. The percentage of CD35 + monocytes in the partial 1-year treatment response group was 23.2% (SD, 3.6), significantly lower than 25.1% (SD, 3.9) of good 1-year treatment response (*P* = 0.039). The percentage of CD35 + classical monocytes in the partial 1-year treatment response group was 25.2% (SD, 4.0), significantly lower than 27.9% (SD, 4.9) in the good 1-year treatment response group (*P* = 0.019). There was no significant difference between poor and good 1-year responders in the proportions of CD35 + monocytes or CD35 + classical monocytes. Neither were there any significant differences between other Cregs in the 1-year treatment response groups (Fig. 8). There was a tendency of a lower proportion of CD46 + non-classical monocytes in the partial response group, however not significant (*P* = 0.29).

**Fig. 9:**
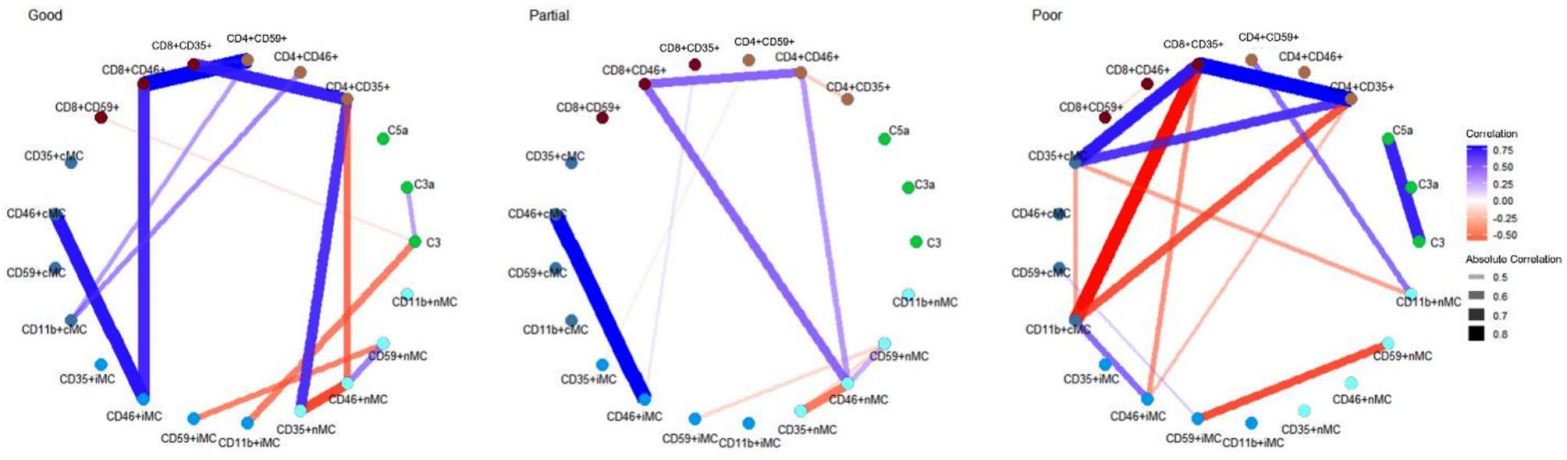
Correlation network of complement proteins and complement regulatory proteins according to 1-year treatment response in neovascular AMD patients, showing statistically significant correlations (*P* < 0.05) with a threshold of absolute correlation coefficient > 0.4

The correlation networks seemed to differ uniquely between 1-year treatment response groups. The partial 1-year treatment response group was the simplest, while good and poor were more complex, in contrast to the correlation networks in the initial treatment response groups (Fig. 9).

### Complement proteins and Cregs Association to genotypes in nAMD patients

Complement protein C3a and the C3a/C3-ratio were significantly elevated in nAMD patients carrying the high-risk CFH rs1061170 genotypes (Table 3). The proportion of CD35 + CD8 + T cells and CD46 + classical monocytes were significantly elevated in high-risk genotypes, while the proportion of CD46 + non-classical monocytes was significantly lower in the high-risk genotypes (Table 3). There were no significant differences in complement proteins or Cregs according to ARMS2 rs10490924 genotype (Table 4).

**Table 3:** Complement proteins and complement regulatory proteins stratified according to CFH rs1061170 genotype

	CFH, rs1061170		
Complement proteins, mean (SD)	CC/CT (high risk), *n* = 82	TT (low risk), *n* = 18	P value
C3 [µg/ml]	876 (398)	800 (243)	0.58
C3a [ng/ml]	573 (251)	435 (111)	**0.0072**
C5a [ng/ml]	27.09 (11.07)	26.10 (14.65)	0.60
C3a/C3-ratio [%]	0.074 (0.037)	0.058 (0.018)	**0.011**
Complement regulatory proteins, mean (SD)			
CD35 + CD4 + T cells [%]	4.47 (1.83)	3.60 (1.05)	0.071
CD35 + CD8 + T cells [%]	6.75 (2.98)	4.96 (3.58)	**0.024**
CD35 + Monocytes [%]	24.26 (3.82)	25.60 (4.15)	0.27
CD35 + Classical monocytes [%]	26.54 (4.47)	29.41 (5.32)	0.077
CD35 + Intermediate monocytes [%]	22.01 (9.04)	19.16 (6.78)	0.28
CD35 + Non-classical monocytes [%]	9.74 (8.26)	6.67 (4.28)	0.27
CD46 + CD4 + T cells [%]	96.94 (2.00)	97.19 (1.72)	0.61
CD46 + CD8 + T cells [%]	98.82 (2.30)	98.43 (2.68)	0.60
CD46 + Monocytes [%]	99.12 (0.96)	99.01 (0.53)	0.52
CD46 + Classical monocytes [%]	99.76 (0.26)	99.54 (0.38)	**0.043**
CD46 + Intermediate monocytes [%]	99.87 (0.26)	99.49 (0.70)	0.050
CD46 + Non-classical monocytes [%]	92.21 (9.03)	94.76 (3.20)	**0.043**
CD59 + CD4 + T cells [%]	92.70 (5.34)	91.99 (7.15)	0.68
CD59 + CD8 + T cells [%]	67.05 (8.76)	71.10 (10.27)	0.16
CD59 + Monocytes [%]	48.09 (5.29)	48.77 (1.85)	0.27
CD59 + Classical monocytes [%]	51.12 (2.53)	51.43 (2.12)	0.59
CD59 + Intermediate monocytes [%]	26.95 (17.91)	26.19 (16.65)	0.98
CD59 + Non-classical monocytes [%]	45.92 (14.43)	48.46 (14.47)	0.28
CD11b + Monocytes [%]	98.04 (0.68)	97.81 (0.94)	0.36
CD11b + Classical monocytes [%]	97.63 (0.84)	97.31 (1.26)	0.33
CD11b + Intermediate monocytes [%]	99.69 (0.48)	99.84 (0.25)	0.065
CD11b + Non-classical monocytes [%]	99.83 (0.24)	99.88 (0.14)	0.19

Bold values indicate statistical significance (*P* < 0.05)

**Table 4:** Complement proteins and complement regulatory proteins stratified according to ARMS2 rs10490924 genotype

	ARMS2, rs10490924		
Complement proteins, mean (SD)	TT/TG (high risk), *n* = 45	GG (low risk), *n* = 55	P value
C3 [µg/ml]	837 (409)	893 (330)	0.32
C3a [ng/ml]	509 (216)	597 (257)	0.057
C5a [ng/ml]	26.38 (12.16)	27.58 (11.25)	0.62
C3a/C3-ratio [%]	0.067 (0.028)	0.076 (0.041)	0.30
Complement regulatory proteins, mean (SD)			
CD35 + CD4 + T cells [%]	4.15 (1.39)	4.54 (2.11)	0.51
CD35 + CD8 + T cells [%]	6.28 (3.12)	6.66 (3.18)	0.46
CD35 + Monocytes [%]	26.22 (3.64)	24.80 (4.19)	0.54
CD35 + Classical monocytes [%]	26.65 (4.40)	27.47 (5.08)	0.46
CD35 + Intermediate monocytes [%]	20.86 (8.11)	22.37 (9.48)	0.47
CD35 + Non-classical monocytes [%]	7.08 (5.96)	7.71 (6.91)	0.62
CD46 + CD4 + T cells [%]	97.03 (1.78)	96.93 (2.17)	0.81
CD46 + CD8 + T cells [%]	98.84 (1.14)	98.66 (1.33)	0.71
CD46 + Monocytes [%]	99.17 (0.63)	99.02 (0.98)	0.41
CD46 + Classical monocytes [%]	99.75 (0.22)	99.69 (0.27)	0.28
CD46 + Intermediate monocytes [%]	99.82 (0.18)	99.78 (0.12)	0.60
CD46 + Non-classical monocytes [%]	92.92 (5.97)	92.29 (7.71)	0.59
CD59 + CD4 + T cells [%]	93.22 (4.14)	91.77 (7.09)	0.21
CD59 + CD8 + T cells [%]	68.12 (9.32)	67.26 (8.90)	0.70
CD59 + Monocytes [%]	48.46 (3.24)	47.90 (6.39)	0.47
CD59 + Classical monocytes [%]	50.96 (2.47)	51.44 (2.43)	0.35
CD59 + Intermediate monocytes [%]	24.80 (17.34)	29.33 (17.86)	0.071
CD59 + Non-classical monocytes [%]	49.06 (13.46)	42.99 (14.95)	0.22
CD11b + Monocytes [%]	98.03 (0.68)	97.97 (0.80)	0.66
CD11b + Classical monocytes [%]	97.62 (0.89)	97.53 (0.97)	0.65
CD11b + Intermediate monocytes [%]	99.65 (0.34)	99.79 (0.21)	0.11
CD11b + Non-classical monocytes [%]	99.82 (0.22)	99.86 (0.23)	0.42

## Discussion

This prospective cohort study aimed to analyze differences in plasma concentration of complement proteins and proportions of membrane Cregs on T cells and monocytes in patients with nAMD and iAMD compared to healthy controls. The association between complement proteins, Cregs and treatment response in nAMD was also evaluated at post-loading dose and after one year, as well as genotypes of CFH and ARMS2 in nAMD patients.

In this study we found that circulating C3 was significantly elevated in nAMD and iAMD patients compared to healthy controls. C3a and the C3a/C3-ratio was also significantly elevated in nAMD patients compared to healthy controls. This suggests an elevated activation of the complement system in patients with AMD, and especially in nAMD patients. Elevated levels of C3 and C3a are associated with increased systemic inflammation by facilitating opsonization, leading to phagocytosis, recruitment of inflammatory cells and antibodies [35, 36]. We did not find C5a to be elevated in iAMD og nAMD patients. A previous study investigating C3 and C5a levels in nAMD patients found a significantly elevated concentration of C5a compared to healthy controls, but did not find any difference in C3 concentrations [24]. Another study investigating C3a levels found patients with nAMD and dry AMD, including both intermediate AMD and geographic atrophy, had significantly elevated levels of C3a compared to a control group [13]. In studies not differentiating between AMD phenotypes compared to healthy controls, C3a and C5a in AMD patients was found significantly increased, while C3 was non-significantly different in the AMD group in one study [11], and C5a significantly increased, while C3 and C3a were non-significantly different in another study [10]. These differing results might be attributed to the different complement measurement protocols. Increased complement activation has also been found in patients with geographic atrophy [25, 37, 38]. The C3 inhibitor pegcetacoplan and C5 inhibitor avacincaptad pegol has been FDA-approved as they are shown to significantly halt the atrophy progression in patients with geographic atrophy secondary to AMD. These drugs are administered intravitreally, and a systemic treatment has yet to be approved [39, 40], but indicates that the complement system might be an important target for AMD in general. The elevated C3a/C3-ratio in nAMD patients suggests increased activation of the complement system, as C3 is converted to C3a by the C3 convertase upon activation [16]. Other ratios between a C3 activation fragment and C3 can be used as an estimation of complement activation. Multiple studies have found an association between C3d/C3-ratio and AMD [28, 41] and specifically nAMD [24], which is comparable to the findings of this current study. The role of C3 in nAMD has been demonstrated in C3 knockdown mice, that did not develop neovascularizations after laser photocoagulation [42], and local C3a increase on inducing neovascularization after laser photocoagulation in mice [43]. C3 has also been found in histological specimens of choroidal neovascularizations in human nAMD patients [44].

The proportion of CD46 + CD4 + T cells and CD59 + intermediate monocytes were significantly decreased in nAMD patients compared to healthy controls. CD46 has an important role as an inactivator of the central complement fragments C3b and C4b [45]. The decreased proportion of CD46 + CD4 + T cells in nAMD patients could cause a dysregulation of the complement cascade, as these patients will have an increased activation of the complement system, including C3 and C3a, leading to phagocytosis and inflammation [46]. Although the difference was slight, AMD is a disease characterized by chronic low-grade inflammation, and these slight changes present during many years might be part of the cause [47–50]. CD46 + CD4 + T cells have also been shown to be important in regulation of inflammation, producing the anti-inflammatory cytokine interleukin-10 [51]. CD59 inhibits the activation of MAC and nAMD patients with decreased CD59 + intermediate monocytes might thus have an increased activation of MAC, causing cell lysis [52]. Intermediate monocytes play an important proinflammatory role, although not yet fully elucidated [53]. This dysregulation of the complement system and the increased complement proteins systemically could cause increased inflammation manifested in the retina [9]. Retinal inflammation leads to tissue damage causing the development of drusen and degeneration of the blood-retinal-barrier consisting of Bruch’s membrane, the retinal pigment epithelium (RPE) and microvascular endothelium. With this degeneration, oxygenation of the retina might be compromised and the rescue mechanism of macular neovascularization can come into effect [54, 55]. Also, the transportation of waste products might be compromised causing debris to accumulate, forming drusen [56]. The complement proteins and Cregs C3, C3a, C5a, CD35 and CD46 have been found in drusen, while CD59 + RPE cells was found reduced overlying drusen in immunohistochemical analyses [57–60]. A previous study also found that the proportion of CD46 + and CD59 + leukocytes were lower in nAMD patients compared to healthy controls [18]. We were not able to replicate the findings of Haas et al., who reported that patients with nAMD had a significantly higher proportion of CD35 + leukocytes compared to healthy controls [19]. Neither did we find increased proportions of CD11b + monocytes in nAMD patients compared to healthy controls reported by Subhi et al. [17]. This may be due to differences in the flow cytometry protocol and gating strategy of the blood samples.

To our knowledge, this is the first study to investigate complement proteins and Cregs in association to treatment response in nAMD. Since the introduction of intraocular anti-VEGF injections, the prevalence of blindness caused by nAMD has significantly decreased. A clinical challenge is however, that many patients respond partially or poorly to this treatment, the reason being largely unknown. We hypothesized that the neovascularization in these patients is mediated by a different signal than ocular VEGF, which might be a dysregulated complement system. Thus, we expected the concentrations of systemic complement proteins to be increased and Creg expression levels to be decreased in patients with partial and poor treatment response compared to good responders. Surprisingly, in this study we find that systemic C3a and C5a were significantly decreased at baseline in nAMD patients with a partial initial (post-loading dose) treatment response compared to a good response. This might suggest an alternative mechanism, possibly indicating dysregulation and subsequent depletion of complement proteins. This depletion could impair the immune system’s ability to manage inflammation and repair tissue effectively, contributing to the persistence and progression of nAMD despite treatment.

We did however find nAMD patients with partial 1-year treatment response had lower proportion of CD35 + classical monocytes and total monocytes at baseline compared to good responders, in agreement with our hypothesis. CD35 inhibits the complement cascade by removing opsonized antigens [61]. Thus, patients responding partially to anti-VEFG after one year might have long lasting dysregulation of the complement system causing inflammatory changes in the retina. As systemic low-grade age-related inflammation is known to cause inflammatory tissue damage in multiple diseases, like Alzheimer’s disease, chronic kidney disease, cardiovascular diseases, and diabetes mellitus [9, 62, 63], so can chronic activation and dysregulation of the complement system cause retinal damage leading to AMD [64]. Patients responding partially to treatment, with altered CD35 expression, might have more low-grade inflammation causing a proinflammatory milieu in the retina, thus not responding ideally [65]. As the statistical tests are adjusted for age, the low-grade inflammation might be a sign of immunosenescence and biological aging, which can be caused by genetics and environmental factors [2, 9, 66–68].

Correlation networks show the phenotypically different interactions between complement proteins and Cregs between healthy controls, iAMD and nAMD patients. Especially iAMD patients seem to have a more complex network than healthy controls and nAMD patients. This might be because iAMD patients are a more heterogeneous group. Patients with iAMD have a 27% risk of developing late-stage AMD within 5 years, while a large proportion never develops late AMD [69]. In this group, there might thus be patients with the same immunological phenotype as nAMD patients that have not yet developed nAMD, as well as geographic atrophy, which might have yet another unique correlation network. Part of the iAMD group might also have a specific relationship not similar to late-stage profiles. For initial treatment response in nAMD patients, it seems that complement proteins play a more central role in poor and partial responders. Furthermore, the partial group seems to have the most complex correlation network, which like iAMD might be caused by being a mixture of potential good and poor late responders. Complement proteins seemed to play a more central role in partial and poor responders compared to good responders. As the correlation networks suggest after the 1-year treatment response, the partial group seems to have simplified, as they may be settled in their final treatment response group after one year, while good and poor 1-year networks have become more complex, as a result of less extreme phenotypes, that takes longer to settle in treatment outcomes. This is similar to a study investigating the correlation networks in nAMD treatment response of chemokine receptors [70].

Genetic susceptibility plays a major role in development of AMD. The two main risk SNPs are the CFH rs1061170 and ARMS2 rs10490924 [24–29]. CFH acts as an important regulator of the complement system controlling the alternative pathway and accelerating the decay of this pathways C3 convertase [66, 71]. Decreased levels of CFH will lead to failure to downregulate the spontaneous activation of C3 [72]. ARMS2 polymorphisms has been suggested to be involved in the activation of the complement system with a genetic interaction between CFH and ARMS2 in AMD patients [24], although the exact function of the ARMS2 protein is yet to be determined [29]. We find that the concentration of C3a was elevated in nAMD patients carrying the high-risk CFH rs1061170 genotype. The C3a/C3-ratio was also elevated in these high-risk carriers, which is in agreement with the previous studies, that find the C3d/C3-ratio was associated with the high-risk alleles of this CFH SNP [24, 41]. Thus, the increased complement activation of nAMD patients could be explained by the CFH genotype associated with nAMD development, which is also demonstrated previously [11, 73]. In this study, we found that a decreased expression level of CD46 + non-classical monocytes in nAMD patients with the high-risk CFH genotypes, corresponding to the increased complement activation. Surprisingly, we found increased expression levels of CD46 + classical monocytes and CD35 + CD8 + T cells which would suggest a higher complement regulation in these patients. The regulatory properties of these particular cell types might be complex and interlinked [65]. We did not find any association between ARMS2 genotype and complement proteins or Cregs. A previous study did not find an association between C3 or C3a and ARMS2 but did find an association with C5a in a population of AMD patients and healthy controls [10].

As nAMD patients respond differently to treatment, planning an individualized treatment strategy might improve visual outcomes for these patients. Predicting the response is essential in such a planning process, and determining the individual profile of circulating complement proteins and Cregs might be useful in such predictions. Furthermore, the complement system and Cregs may possibly be targets for new therapeutics in addition to the current intraocular anti-VEGF treatment. It might be beneficial for patients with iAMD and high risk of developing late AMD [74] to be treated before occurrence of these vision impairing states. C3 could potentially be a target for this treatment. However, the elevated complement proteins and decreased Creg levels were associated with partial treatment response within the nAMD group, and a complement inhibitory treatment might cause potential good responders to respond partially. Patients with iAMD treated this way might also be at risk of becoming partial responders. The dysregulation of the complement system is complex and influenced by genetic factors in patients with AMD.

Limitations of this study include the observational study design, which precludes any definitive conclusions about causality. Furthermore, the relatively small number of nAMD patients with poor response might have hidden significant correlations. The categorical nature of the treatment response classification is a limitation, as especially the partial response group includes all patients with decreased CRT and persistent retinal fluid. Thus, patients with major quantities of retinal fluid at baseline, that have persistence of minor cysts will still be in the partial group. There was a significant age difference between the groups, leading to all ANCOVA analyses being adjusted for age, although age-matched groups would have been more ideal. The EDTA coated tubes for immunoassays were centrifuged at 20 °C, rather than 4 °C, which might have led to some complement activation [75].

In conclusion, patients with nAMD and iAMD have elevated levels of complement proteins, and nAMD have decreased levels of Cregs compared to healthy controls. Patients with nAMD, who respond partially to anti-VEGF treatment have a dysregulation of the complement system and Cregs.

## Electronic supplementary material

Below is the link to the electronic supplementary material.

Supplementary Material 1

## Data Availability

No datasets were generated or analysed during the current study.
