# Supplementary material for: Complement proteins and complement regulatory proteins are associated with age-related macular degeneration stage and treatment response
Source: J Neuroinflammation. 2024 Nov 1;21:284. doi: 10.1186/s12974-024-03273-7 (PMC11531117; doi:10.1186/s12974-024-03273-7)
Supplement: Supplementary file 1 — Supplementary Material 1 [file 12974_2024_3273_MOESM1_ESM.docx]

**Additional File 1**

**Supplementary Table 1**. Flow Cytometry Antibodies.

|  | **CD4** | **CD8** | **CD14** | **CD16** | **CD35** | **CD46** | **CD59** |
| --- | --- | --- | --- | --- | --- | --- | --- |
| **Fluorochrome** | PerCP | Brilliant Violet 510 | Pacific Blue | APC/Cy7 | APC | PE/Cy7 | FITC |
| **Manufacturer** | R&D Systems | BioLegend | BioLegend | BioLegend | R&D Systems | BioLegend | BioLegend |
| **Catalog number** | FAB3791C-100 | 301048 | 325616 | 302018 | FAB5748A | 352408 | 304706 |
| APC= Allophycyanin, APC/Cy7 = Allophycocyanin-cyanine 7, FITC = Fluorescein isothiocyanate, PE/Cy7 = Phycoerythrin-cyanine 7, PerCP = Peridinin-chlorophyll-protein.  R&D Systems, Minneapolis, MN, USA.  Biolegend, San Diego, CA, USA. | | | | | | | |

**Supplementary table 2**. Immunoassays for complement components.

|  | **C3** | **C3a** | **C5a** |
| --- | --- | --- | --- |
| **Immunoassay** | R-plex Human Complement C3 Assay | R-plex Human Complement C3a Assay | R-plex Human Complement C5a Assay |
| **Catalog number** | K151XYR-2 | K151V0R-2 | K151K4R-2 |
| Manufacturer: Mesoscale Discovery, Rockville, MD, USA | | | |
